# Depressive symptoms and Learning Motivation among Chinese freshmen: a longitudinal study using random intercept cross-lagged panel model and the subgroup analysis of parental educational background

**DOI:** 10.3389/fpsyt.2025.1715472

**Published:** 2025-12-05

**Authors:** Ye Yu, Ting Yu, Xinfeng Zhang, Li Zhang, Xuejian Su, Xinwei Yu, Yuwei Yang, Ting Pan, Xiaopeng Deng

**Affiliations:** 1 Mental Health Center of Yangtze University, Jingzhou, Hubei, China; 2 Mental Health Institute of Yangtze University, Jingzhou, Hubei, China; 3 Jingzhou Rongjun Special Care Hospital, Jingzhou, Hubei, China; 4 Jingzhou Mental Health Center, Jingzhou, Hubei, China; 5 Jingzhou Hospital of Traditional Chinese Medicine, Jingzhou, Hubei, China

**Keywords:** college freshmen, depressive symptoms, learning motivation, parental educational background, random intercept cross-lagged panel model, longitudinal study

## Abstract

**Objectives:**

This study aimed to explore the bidirectional association between depressive symptoms and learning motivation (LM) among Chinese college freshmen, distinguish between interindividual and intraindividual effects, and analyze the moderating role of parental educational background.

**Methods:**

A prospective longitudinal study was conducted among freshmen from a university in Jingzhou City, Hubei Province, China, with three waves of surveys over two years (2023–2025) and follow-ups conducted annually. Data from a total of 4,669 freshmen were finally included in the analysis (mean age = 19.32 years, SD = 2.96; 53.8% female). Depressive symptoms were assessed using the Patient Health Questionnaire-9 (PHQ-9), and LM was measured via the LM Subscale of the Mental Health Scale for College Students (MHS-CS). The Random Intercept Cross-Lagged Panel Model (RI-CLPM) was employed to analyze the temporal association between depressive symptoms and LM, and separate models were constructed for the full sample, samples from low-educational background families, samples from intermediate-education background families, and samples from high-educational background families.

**Results:**

At the interindividual level, all groups showed a strong negative correlation between depressive symptoms and LM (full sample: r=-0.70, p<0.001; low-education families: r=-0.66, p<0.001; intermediate-education families: r=-0.72, p<0.001; high-education families: r=-0.85, p<0.001). At the intraindividual level, depressive symptoms were significantly negatively correlated with LM across all waves in all groups (r=-0.30~-0.46). For cross-lagged effects: depressive symptoms stably and negatively predicted subsequent LM at T1→T2 (β=-0.12) and T2→T3 (β=-0.10~-0.23) in all groups; LM negatively predicted depressive symptoms only at T1→T2 in the full sample (β=-0.11) and low-education families (β=-0.12), positively predicted depressive symptoms at T2→T3 in intermediate-education families (β=0.08), and had no significant effect across all waves in high-education families.

**Conclusions:**

A stable negative correlation exists between depressive symptoms and LM among Chinese college freshmen: depressive symptoms consistently impair subsequent LM, while the protective effect of LM against depressive symptoms is phased, with subgroup differences in family educational background. These findings suggest that colleges and universities should prioritize early screening and intervention for freshmen’s depressive symptoms to protect their LM, and provide targeted support based on parental educational background.

## Introduction

Depressive symptoms have become increasingly prevalent in modern society. Characterized by low mood, reduced anhedonia, and decreased energy, depression is a severe mental health issue (1). The World Health Organization predicts that by 2030, depression may surpass cardiovascular and respiratory diseases to become a major cause of global disease burden (2).

The status of depressive symptoms among college freshmen is particularly concerning, as various studies have reported a high detection rate of depressive symptoms in this population. One study found that 41.4% of college freshmen exhibited depressive symptoms, with 23.6% testing positive for depression (3). Another study reported that the rate of college students with depressive symptoms was as high as 59.8%, varying in severity (4). These alarming findings highlight the need for continuous attention to depressive symptoms among college freshmen.

LM drives students’ engagement in the educational process through the complex interaction of internal and external factors (5). Intrinsic motivation stems from personal interest and inherent satisfaction with learning, while extrinsic motivation is influenced by external rewards or pressures (6). Understanding the factors affecting LM is crucial for improving the quality of higher education and supporting student development (7).

Depressive symptoms exert a profound impact on LM, as evidenced by multiple studies. Research has indicated that depression significantly impairs intrinsic LM (8). Among college students, there is a significant negative correlation between depressive symptoms and LM, meaning increased depressive symptoms are associated with decreased LM (9). Other studies have emphasized that depressive symptoms negatively affect students’ academic performance and LM, and mental health interventions can enhance students’ LM (10).

Conversely, LM also plays a vital role in influencing depressive symptoms. Positive LM is a crucial protective factor against depressive symptoms. Studies have shown that higher motivation predicts a reduction in depression after one year (11). Therefore, fostering LM may serve as an effective pathway to enhance mental health. Furthermore, compared with other health-related risk factors, positive motivation has greater predictive value for mental health status (11). Additional research has confirmed that enhancing motivation can also positively influence neurocognitive function in individuals with depression (12).

Notably, parental educational background appears to be involved in the relationship between LM and depressive symptoms. Studies have shown that parental educational level is positively correlated with children’s LM (13). In addition, the overall educational level of parents is negatively correlated with depressive symptoms in adolescents; higher parental educational level may help reduce adolescents’ depressive symptoms (14). A meta-analysis involving adolescents from 10 countries found a significant negative correlation between parental educational level and adolescent depression (15). These findings are consistent with the core tenets of family systems theory, which holds that interactions and influences exist among family members (16). Family educational background is not an isolated demographic characteristic; instead, it significantly shapes the family environment, affecting access to educational resources, choices of parenting styles, and provision of emotional support (17). This set of factors may, in turn, shape students’ LM, reduce the risk of depression, and ultimately become an important variable influencing the association between LM and depressive symptoms.

The traditional Cross-Lagged Panel Model (CLPM) is primarily used to explore longitudinal correlations between variables, but it relies on a critical assumption: there are no stable intraindividual differences between variables, and only interindividual effects exist (18). This assumption is often difficult to validate in real-world research. Existing studies have confirmed that confounding interindividual effects (differences between individuals) with intraindividual effects (changes within an individual over time) may lead to biased estimates of cross-lagged effects, including overestimation, underestimation, or even reversal of effect direction (19). Therefore, conclusions drawn by CLPM at the interindividual level cannot be directly generalized to the dynamic intraindividual relationships (20). For example, observing that “college freshmen with depressive symptoms generally have lower LM than other students” at the interindividual level does not equate to “improved depressive symptoms in a specific freshman will necessarily enhance their own LM” at the intraindividual level. In contrast, the Random Intercept Cross-Lagged Panel Model (RI-CLPM) introduces a random intercept to account for time-invariant individual differences, thereby clearly separating interindividual and intraindividual effects in the longitudinal associations between variables. For the estimation of intraindividual effects, RI-CLPM has significantly smaller errors than the traditional CLPM and can more accurately reflect the dynamic interaction of variables within an individual over time (19).

For college freshmen, LM is a core issue related to their adaptation and development; thus, an increasing number of studies have explored the association between LM and mental health. However, the direction and specific mechanisms of their interaction remain unclear. Clarifying this relationship holds important theoretical and practical value for optimizing mental health support and academic guidance strategies for college freshmen. Against this background, the purpose of this study was to use RI-CLPM to examine the longitudinal bidirectional relationship between depressive symptoms and LM among college freshmen at both the interindividual and intraindividual levels, and to explore the moderating role of parental educational background.

## Methods

### Participants

**Figure 1:**
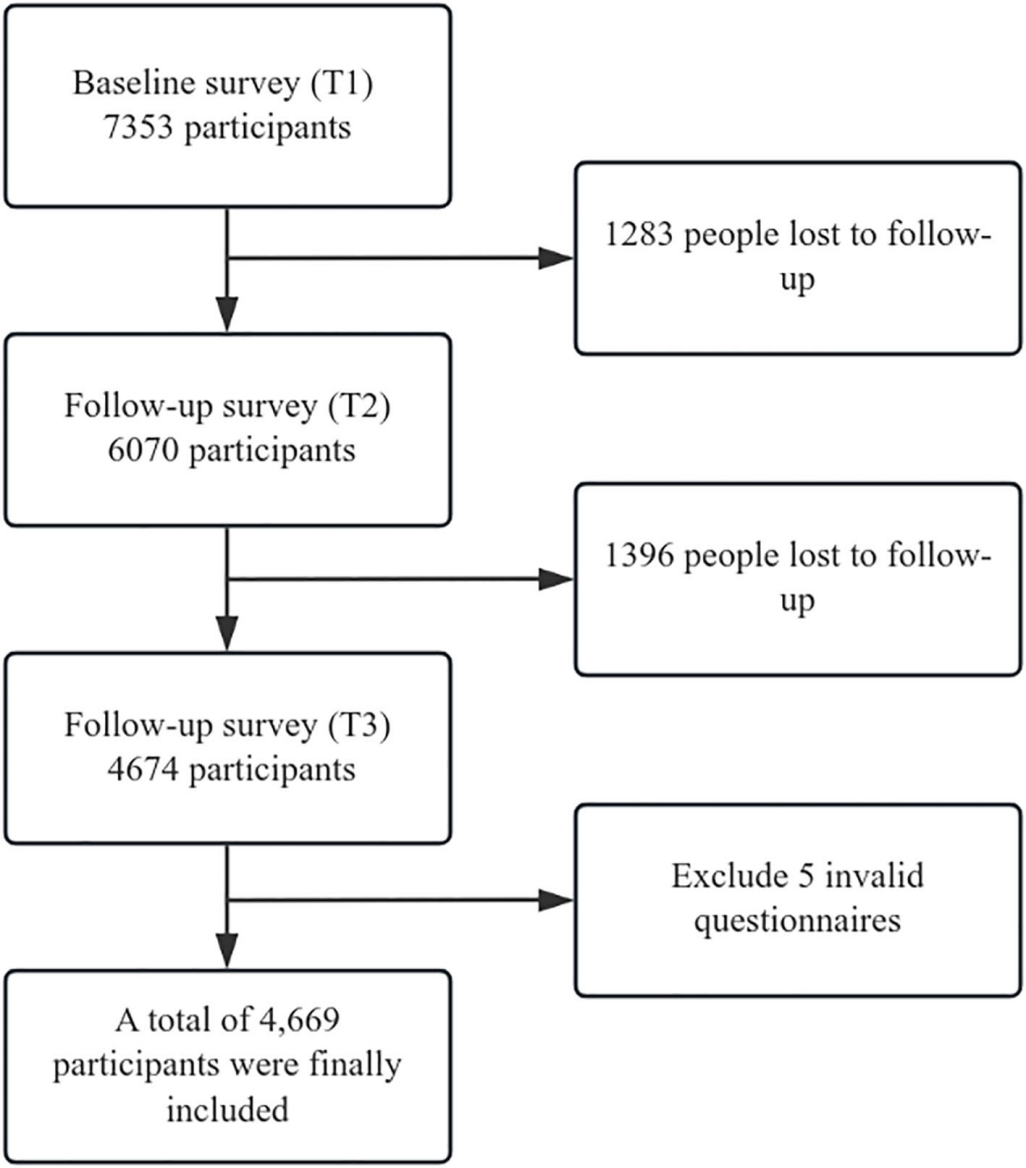
Data flow diagram.

This study recruited freshmen from a university in Jingzhou City, Hubei Province, China. A longitudinal follow-up design was adopted, with three waves of self-reported surveys (T1–T3) conducted over two years from 2023 to 2025. A total of 7,353 freshmen were included in the baseline survey (T1), with a mean age of 19.32 years (SD = 2.96). At T2 and T3, 6,070 and 4,674 participants completed the follow-up surveys, with retention rates of 82.55% and 63.57%, respectively. We used the regression estimation method to handle missing data.Attrition was mainly attributed to objective factors such as students’ participation in off-campus internships. Among the questionnaires collected at T3, 5 with missing partial information were excluded, and 4,669 participants were finally included in the analysis (**Figure 1**).

To assess the possibility of systematic attrition bias, we used the Mann-Whitney U test to compare parental educational background, PHQ-9 scores, and LM scores at the baseline (T1) stage between retained participants (n=4,669) and withdrawn participants (n=2,684). The results showed no significant differences in father’s educational level (Z=-0.445, p=0.656), mother’s educational level (Z=-0.490, p=0.624), PHQ-9 scores (Z=-0.412, p=0.680), and LM scores (Z=-0.851, p=0.395). This indicates that attrition is not related to key variables, and the risk of systematic bias is low.

### Procedure

In September 2023 (T1), the first questionnaire was distributed, and participants signed a confidentiality agreement for data use. The data provided by participants were handled properly to ensure the protection of their privacy. At the beginning of the questionnaire, students were informed that the study focused on LM, depressive symptoms, etc., and that participation might take time without direct benefits but was meaningful for academic research. Students were given the right to voluntarily decide whether to participate after fully understanding the study purpose, and were clearly informed that they could withdraw from the study at any time without any adverse consequences. The director of the university’s psychological counseling center and full-time psychology teachers were responsible for sending the survey protocol to counselors of selected colleges and providing them with unified training. Counselors then sent the questionnaire link to students via WeChat. Each student was required to carefully read the instructions on the study purpose and questionnaire completion method. All students were aware of the study purpose, and voluntary participation was deemed as informed consent. This study was approved by the Ethics Committee of Jingzhou Mental Health Center. Data for T2 and T3 were collected approximately 1 year and 2 years later, respectively.

### Measures

#### Demographic information

Self-reported demographic information included gender, family economic status, and parental educational level. Based on previous studies, gender and family economic status were included as covariates (21, 22). Family economic status was coded as 1 (poor), 2 (ordinary), and 3 (wealthy). Since the focus lies on the perception of relative economic status, this study assesses family economic conditions through the subjective judgments reported by participants. Parental educational level (father and mother separately) was coded as 1 (junior high school or below), 2 (senior high school), and 3 (bachelor’s degree or above). To stratify the population, we categorized and counted parental educational attainment. Families where both parents had an educational attainment of junior high school or below were classified as low-education families; families where one parent had a bachelor’s degree or above and the other had junior high school or below or senior high school education, or both parents had senior high school education were classified as intermediate-education families; families where both parents had a bachelor’s degree or above were classified as high-education families.

#### Patient health questionnaire-9

The PHQ-9 was developed based on the diagnostic criteria for depression in the Diagnostic and Statistical Manual of Mental Disorders, Fourth Edition (DSM-IV) to assess depressive symptoms over the past two weeks (23). The questionnaire consists of 9 items, each rated on a 4-point scale ranging from 0 (not at all) to 3 (nearly every day). The total score ranges from 0 to 27, with higher scores indicating more severe depressive symptoms. The Chinese version of the PHQ-9 has good reliability and validity (24). In this study, Cronbach’s α coefficients for the depressive symptom scale at T1–T3 were 0.87, 0.89, and 0.90, respectively.

#### Learning motivation

LM was assessed using the LM Subscale of the Mental Health Scale for College Students (MHS-CS), developed by Huang Xiting, to evaluate college students’ attitudes toward learning and experiences during the learning process (25, 26). This questionnaire includes 5 items, each rated on a 5-point Likert scale ranging from 1 (strongly agree) to 5 (strongly disagree). The total score ranges from 5 to 25, with higher scores indicating stronger active learning ability (26). In this study, Cronbach’s α coefficients for the LM scale at T1–T3 were 0.85, 0.87, and 0.89, respectively.

### Data analysis strategy

First, descriptive analysis and correlation analysis were conducted. Second, the intraclass correlation coefficients (ICC) of LM and depressive symptoms were calculated. A high ICC (> 0.50) indicates that between-individual variance is dominant, while a low ICC (< 0.50) indicates that within-individual variance is dominant (27).

Third, Confirmatory Factor Analysis (CFA) was used to test longitudinal measurement invariance, examining the equivalence of LM and depressive symptoms across different time points to ensure that observed effects were attributed to real changes in variables. A three-step procedure was adopted for measurement invariance, including configural, metric, and scalar invariance (28).

Finally, RI-CLPM was used to test the bidirectional relationship between LM and depressive symptoms. Gender and family economic status of freshmen were included as time-invariant covariates, and all variables were regressed at the three time points. Four RI-CLPM models were constructed in this study: the first model examined the relationship between LM and depressive symptoms in all participants; the second model focused on this relationship in low-educational background families; the third model examined the relationship between LM and depressive symptoms in intermediate-education families; and the fourth model explored the relationship in high-educational background families.

For each model, the chi-square value (χ²), Comparative Fit Index (CFI), Standardized Root Mean Square Residual (SRMR), and Root Mean Square Error of Approximation (RMSEA) were reported to evaluate model fit (29, 30). A model was considered to have acceptable fit if CFI > 0.90 and both RMSEA and SRMR < 0.08 (31). To obtain robust estimates, time-invariance tests were performed for all cross-lagged paths, autoregressive paths, and correlated changes to enhance the parsimony of the RI-CLPM. If there was no significant difference in fit between the constrained model and the unconstrained model, the constrained (i.e., more parsimonious) model was retained. CFI and RMSEA were used as the two criteria for model comparison. Two models were considered not significantly different if △CFI < 0.010 and △RMSEA < 0.015 between the constrained and unconstrained models (32). All analyses were performed using SPSS version 27 (Armonk, NY, USA) and Mplus version 8.7 (Muthén and Muthén, Los Ange-les, CA, USA).

## Results

### Common method bias test

Harman’s single-factor test was used to examine common method bias for the three waves of data respectively (33). The results showed that the explanatory rate of the first factor in the T1 stage was 41.17%, 45.67% in the T2 stage, and 46.88% in the T3 stage, all of which were lower than the threshold of 50%. This indicates that there is no serious common method bias in the data of this study.

### Descriptive statistics

**Table 1** presents the demographic characteristics of participants and descriptive statistics of depressive symptom and LM scores. The gender ratio was balanced, and most participants had an ordinary family economic status. Mothers had a slightly lower educational level than fathers, and the proportion of low-educational background families was higher than that of high-educational background families.

**Table 1:** Baseline characteristics of the study sample (n = 4669).

Variables	Category	n (%) or Median (Quartile)
Gender	Men	2157(46.1)
	Women	2512(53.8)
Family economy	Wealthy	122(2.6)
	Ordinary	3442(73.7)
	Poor	1105(23.6)
Educational background		
Father	Junior high school and below	2553(54.6)
	Senior high school	1215(26.0)
	Undergraduate and above	901(19.3)
Mother	Junior high school and below	3004(64.3)
	Senior high school	856(18.3)
	Undergraduate and above	537(17.3)
Parents	Low educational background	2201(47.1)
	High educational background	746(15.9)
Depression T1		3(1, 6)
Depression T2		2(0, 5)
Depression T3		1(0, 4)
Lm T1		19(16, 22)
Lm T2		19(16, 21)
Lm T3		20(16, 22)

Lm, LM.

**Table 2** shows the correlation matrix of all variables in the total sample (n = 4,669). Effect sizes of correlation coefficients were defined based on absolute values: low (r > 0.10), moderate (r > 0.30), and high (r > 0.50) (34). At the same time point, LM was highly negatively correlated with depressive symptoms (-0.530~-0.538). Gender was only significantly correlated with depressive symptoms at T3. Family economic status, father’s educational level, and mother’s educational level were all negatively correlated with depressive symptoms and positively correlated with LM (p < 0.01).

**Table 2:** Correlation matrix (n = 4669).

Variables	1	2	3	4	5	6	7	8	9	10
1.Depression T1	–									
2.Depression T2	.435**	–								
3.Depression T3	.336**	.496**	–							
4.Lm T1	-.538**	-.372**	-.313**	–						
5.Lm T2	-.371**	-.556**	-.360**	.557**	–					
6.Lm T3	-.331**	-.443**	-.530**	.510**	.615**	–				
7.Gender	0.018	0.026	.073**	-0.009	0.026	0.003	–			
8.Family economy	-.074**	-.060**	-.080**	.081**	.062**	.059**	0.013	–		
9.Father’s educational background	-.063**	-.068**	-.075**	.075**	.085**	.093**	-0.014	.242**	–	
10.Mother’s educational background	-.061**	-.077**	-.064**	.098**	.104**	.085**	-0.012	.248**	.616**	–

**p<0.01 Lm, LM.

**Table 3** presents the correlations between LM and depressive symptoms across the three measurement waves in low- and high-educational background families, respectively. At the same time point, LM was highly negatively correlated with depressive symptoms in low-educational background families (-0.529~-0.539), and moderately to highly negatively correlated with depressive symptoms in high-educational background families (-0.490~-0.561).

**Table 3:** Correlation matrix (population stratification).

	1	2	3	4	5	6
1.Depression T1	–	.419**	.295**	-.561**	-.354**	-.270**
2.Depression T2	.408**	–	.443**	-.345**	-.533**	-.354**
3.Depression T3	.338**	.497**	–	-.320**	-.349**	-.490**
4.Lm T1	-.529**	-.335**	-.288**	–	.538**	.497**
5.Lm T2	-.347**	-.539**	-.362**	.527**	–	.606**
6.Lm T3	-.320**	-.453**	-.536**	.491**	.624**	–

*p<0.05 **p<0.01 Lm, LM. Low-educational background families in the lower left corner, Highly educated families in the upper right corner.

In addition, the ICCs of LM and depressive symptoms reported by the total sample were 0.56 and 0.38, respectively; those reported by participants from low-educational background families were 0.55 and 0.37, respectively; and those from high-educational background families were 0.54 and 0.32, respectively. These results indicate sufficient intraindividual variance to use RI-CLPM to investigate changes over time.

### Longitudinal measurement invariance

As shown in **Table 4**, we examined configural, metric, and scalar invariance of depressive symptoms and LM in the longitudinal measurement model. Factor loadings must be time-invariant to achieve at least metric invariance across time (35). Based on previous studies, metric invariance was established if △CFI < 0.010 and △RMSEA < 0.015 compared with the configural model; scalar invariance was established if △CFI < 0.010 and △RMSEA < 0.015 compared with the metric model (36). The results showed no significant differences between the configural invariance model and the metric invariance model for all variables. The achievement of metric invariance for all study variables supports meaningful comparisons across the three measurement waves.

**Table 4:** Model fit and comparison for measurement invariance.

Variable	Models	χ2 (df)	CFI	SRMR	RMSEA	△CFI	△RMSEA
Full sample							
Depression							
	Configural invariance	1740.127(134)	0.920	0.036	0.051	–	–
	Metric invariance	1821.031(143)	0.916	0.049	0.050	-0.004	-0.001
	Scalar invariance	2512.929(152)	0.882	0.073	0.058	-0.034	0.008
Learning motivation							
	Configural invariance	1514.081(87)	0.948	0.027	0.059	–	–
	Metric invariance	1588.703(97)	0.946	0.032	0.057	-0.002	-0.002
	Scalar invariance	2062.547(107)	0.929	0.045	0.063	-0.017	0.006
Low-education families							
Depression							
	Configural invariance	1032.457(134)	0.910	0.040	0.055	–	–
	Metric invariance	1058.531(143)	0.908	0.047	0.054	-0.002	-0.001
	Scalar invariance	1416.785(152)	0.873	0.072	0.061	-0.035	0.007
Learning motivation							
	Configural invariance	808.908(87)	0.946	0.029	0.061	–	–
	Metric invariance	847.886(97)	0.944	0.033	0.059	-0.002	-0.002
	Scalar invariance	1088.19(107)	0.927	0.045	0.065	-0.017	0.006
Intermediate-education families							
Depression							
	Configural invariance	849.420(134)	0.908	0.042	0.056	–	–
	Metric invariance	874.832(143)	0.906	0.046	0.055	-0.002	-0.001
	Scalar invariance	1018.288(152)	0.889	0.054	0.058	-0.017	0.003
Learning motivation							
	Configural invariance	618.297(87)	0.947	0.028	0.060	–	–
	Metric invariance	656.236(97)	0.944	0.036	0.058	-0.003	-0.002
	Scalar invariance	842.304(107)	0.927	0.050	0.063	-0.017	0.005
High-education families							
Depression							
	Configural invariance	433.282(134)	0.909	0.047	0.055	–	–
	Metric invariance	449.806(143)	0.907	0.068	0.054	-0.002	-0.001
	Scalar invariance	548.868(152)	0.880	0.087	0.059	-0.027	0.005
Learning motivation							
	Configural invariance	163.966(87)	0.958	0.036	0.049	–	–
	Metric invariance	175.302(97)	0.957	0.048	0.047	-0.001	-0.002
	Scalar invariance	206.724(107)	0.945	0.054	0.051	-0.012	0.004

CFI, comparative fit index; df, degrees of freedom; RMSEA, root mean square error of approximation; SRMR, standardized root mean square residual. Δ means the difference between two models.

### RI-CLPM between depressive symptoms and LM

**Table 5** presents the model fit and time-invariance tests of the RI-CLPM for depressive symptoms and LM. Based on the principle of model parsimony, in the full-sample model and the low-education family model, the model fit of the unconstrained baseline model (Model 1) was accepted as the final RI-CLPM. In the intermediate-education family model, the model fit of the model with stability paths fixed as time-invariant (Model 3) was acceptable and adopted as the final RI-CLPM. In the high-education family model, the model fit of the model with cross-lagged paths fixed as time-invariant (Model 2) was acceptable and used as the final RI-CLPM.

**Table 5:** Model fit of RI-CLPMs.

Models	χ2 (df)	CFI	SRMR	RMSEA	△CFI	△RMSEA
Full-sample						
Model 1	2.156(1)	1.000	0.003	0.016	–	–
Model 2	25.811(3)	0.998	0.010	0.040	-0.002	0.024
Model 3	31.851(3)	0.997	0.015	0.045	-0.003	0.029
Model 4	30.546(2)	0.997	0.021	0.055	-0.003	0.039
Model 5	77.062(6)	0.993	0.022	0.050	-0.007	0.034
Low-education families						
Model 1	0.171(1)	1.000	0.001	0.000	–	–
Model 2	7.687(3)	0.999	0.008	0.027	-0.001	0.027
Model 3	22.376(3)	0.996	0.021	0.054	-0.004	0.054
Model 4	8.978(2)	0.998	0.017	0.040	-0.002	0.04
Model 5	44.313(6)	0.992	0.028	0.054	-0.008	0.054
Intermediate-education families						
Model 1	5.846(1)	0.999	0.008	0.053	–	–
Model 2	26.112(3)	0.994	0.018	0.067	-0.005	0.014
Model 3	17.709(3)	0.996	0.012	0.053	-0.003	0.000
Model 4	20.163(2)	0.995	0.026	0.073	-0.004	0.020
Model 5	35.131(6)	0.992	0.022	0.053	-0.007	0.000
High-education families						
Model 1	0.269(1)	1.000	0.003	0.000	–	–
Model 2	1.626(3)	1.000	0.007	0.000	0.000	0.000
Model 3	5.435(3)	0.998	0.022	0.033	-0.002	0.033
Model 4	7.239(2)	0.996	0.028	0.059	-0.004	0.059
Model 5	15.600(6)	0.993	0.029	0.046	-0.007	0.046

Model 1 = unconstrained baseline model; Model 2 = model with all cross-lagged paths fixed to be time-invariant; Model 3 = model with all stabil-ity paths fixed to be time-invariant; Model 4 = model with all T2–T3 correlated changes fixed to be time-invariant; Model 5 = model with all cross-lagged paths, all stability paths, and all T2–T3 correlated changes fixed to be time-invariant.

**Figure 2:**
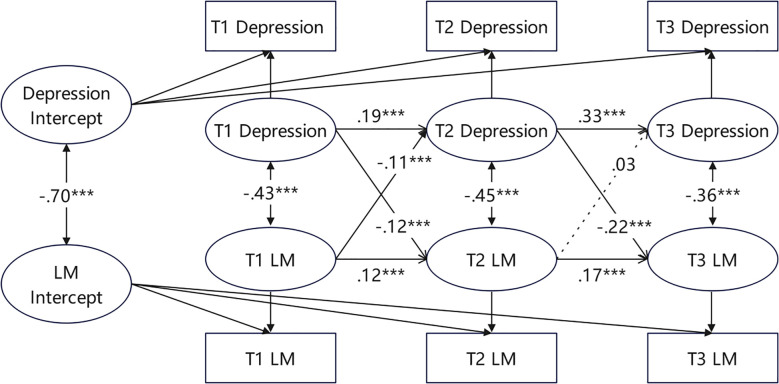
The RI-CLPM of depression and LM across three measurements in the full sample of participants.Standardized estimates, significant (solid lines) and not significant (dotted lines) paths are included. For the sake of clarity, the effects of the covariates (gender and family economic status) are not shown. ***p <.001.

**Figure 2** shows the RI-CLPM between depressive symptoms and LM in the full sample. At the interindividual level, depressive symptoms were highly negatively correlated with LM (r = -0.70, p < 0.001). This means that individuals with consistently low LM exhibited higher levels of depressive symptoms across the three time points, and vice versa. At the intraindividual level, depressive symptoms were also significantly negatively correlated with LM at all three time points (r = -0.36~-0.45, p < 0.001). This indicates that when a college freshman’s LM deviates from their personal baseline at a certain time point, their depressive symptoms are more likely to deviate in the opposite direction. Meanwhile, the autoregressive effects of both depressive symptoms and LM were significant (p < 0.001). This means that if a college freshman’s depressive symptoms or LM are lower than expected at a certain time point, their subsequent depressive symptoms or LM may also be lower than expected. In addition, the intraindividual cross-lagged paths from depressive symptoms to LM were significant at T1-T2 (β = -0.12, p < 0.001) and T2-T3 (β = -0.22, p < 0.001). Conversely, the path from LM to depressive symptoms was significant at T1-T2 (β = -0.11, p < 0.001), but no significant cross-lagged effect was observed at T2-T3. These results suggest that at the intraindividual level, depressive symptoms negatively predicted LM across all three time points, whereas LM negatively predicted depressive symptoms only from T1 to T2.

**Figure 3:**
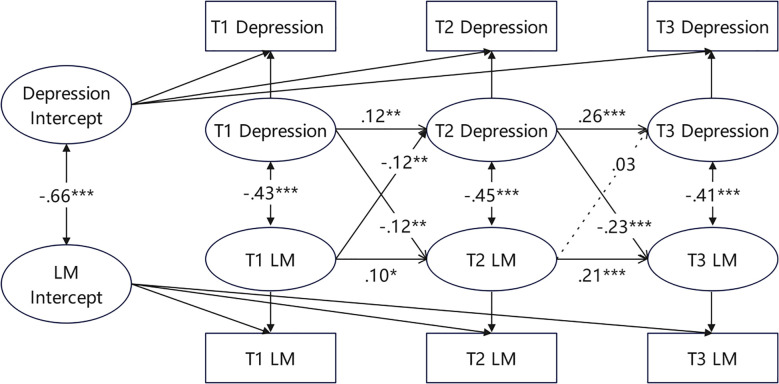
The RI-CLPM of depression and LM across three measurements in families with low educational attainment. Standardized estimates, significant (solid lines) and not significant (dotted lines) paths are included. For the sake of clarity, the effects of the covariates (gender and family economic status) are not shown. *p <.05; **p <.01; ***p <.001.

**Figure 3** shows the RI-CLPM between depressive symptoms and LM in participants from low-educational background families. At the interindividual level, depressive symptoms were negatively correlated with LM (r = -0.66, p < 0.001). At the intraindividual level, depressive symptoms were significantly negatively correlated with LM at all three time points (r = -0.41~-0.45, p < 0.001). The autoregressive effects of both depressive symptoms and LM were significant (p < 0.001). In addition, the intraindividual cross-lagged paths from depressive symptoms to LM were significant at T1-T2 (β = -0.12, p < 0.01) and T2-T3 (β = -0.23, p < 0.001). Conversely, the path from LM to depressive symptoms was significant at T1-T2 (β = -0.12, p < 0.01), but no significant cross-lagged effect was observed at T2-T3. These results indicate that at the intraindividual level, depressive symptoms in low-educational background families negatively predicted LM across all three time points, whereas LM negatively predicted depressive symptoms only from T1 to T2.

**Figure 4:**
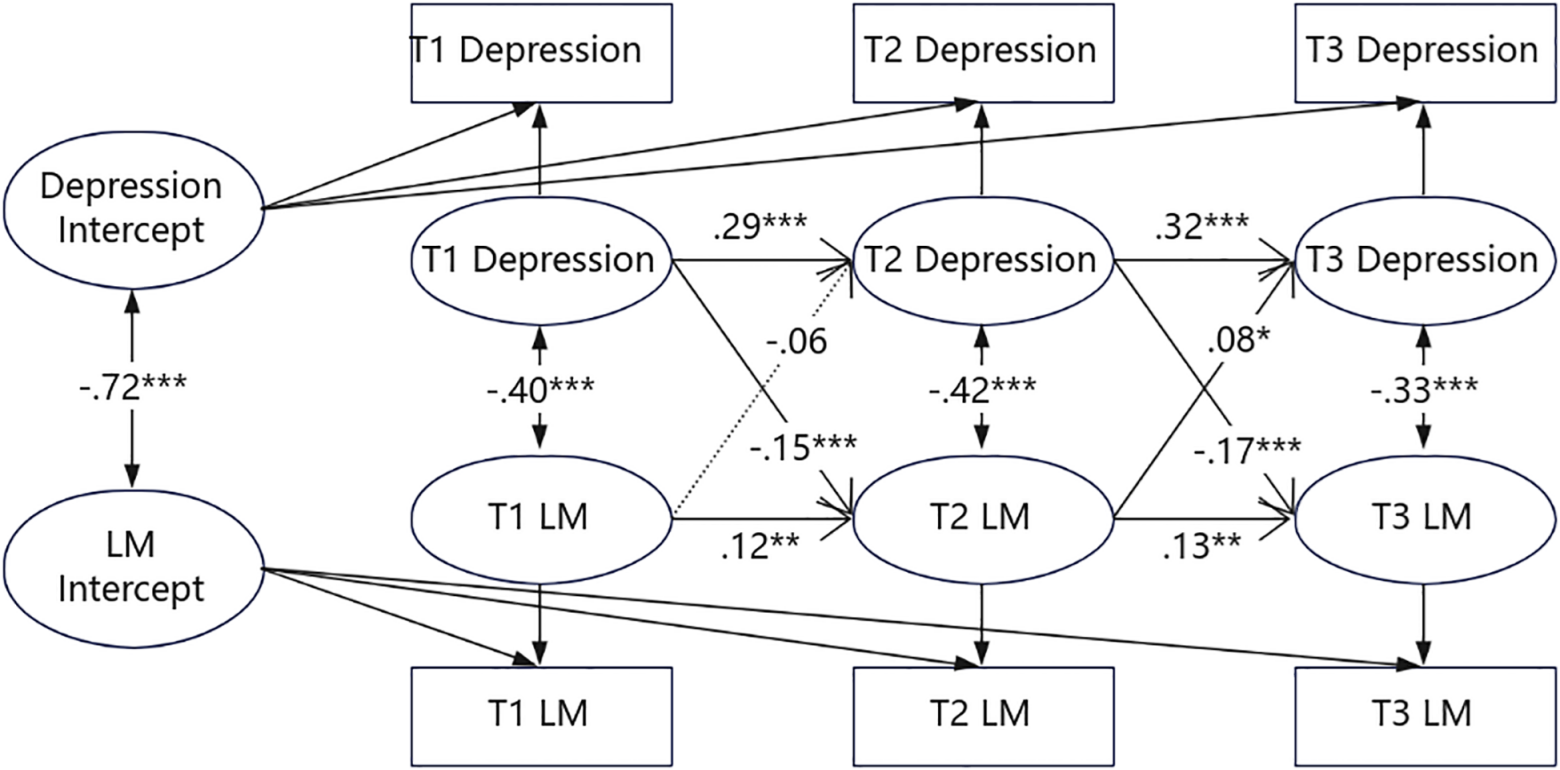
The RI-CLPM of depression and LM across three measurements in families with intermediate educational attainment. Standardized estimates, significant (solid lines) and not significant (dotted lines) paths are included. For the sake of clarity, the effects of the covariates (gender and family economic status) are not shown. *p <.05; **p <.01; ***p <.001.

**Figure 4** shows the RI-CLPM between depressive symptoms and LM among participants from intermediate-education families. At the interindividual level, depressive symptoms were negatively correlated with LM (r = -0.72, p < 0.001). At the intraindividual level, depressive symptoms were significantly negatively correlated with LM at all three time points (r = -0.33~-0.42, p < 0.001). The autoregressive effects of both depressive symptoms and LM were significant (p < 0.01). In addition, the intraindividual cross-lagged paths from depressive symptoms to LM were significant at T1-T2 (β = -0.15, p < 0.001) and T2-T3 (β = -0.17, p < 0.001). Conversely, the path from LM to depressive symptoms was also significant at T2-T3 (β = 0.08, p < 0.05), whereas no significant cross-lagged effect was observed at T1-T2. These results indicate that at the intraindividual level, depressive symptoms in intermediate-education families negatively predicted LM across all three time points; conversely, LM predicted depressive symptoms only from T2 to T3.

**Figure 5:**
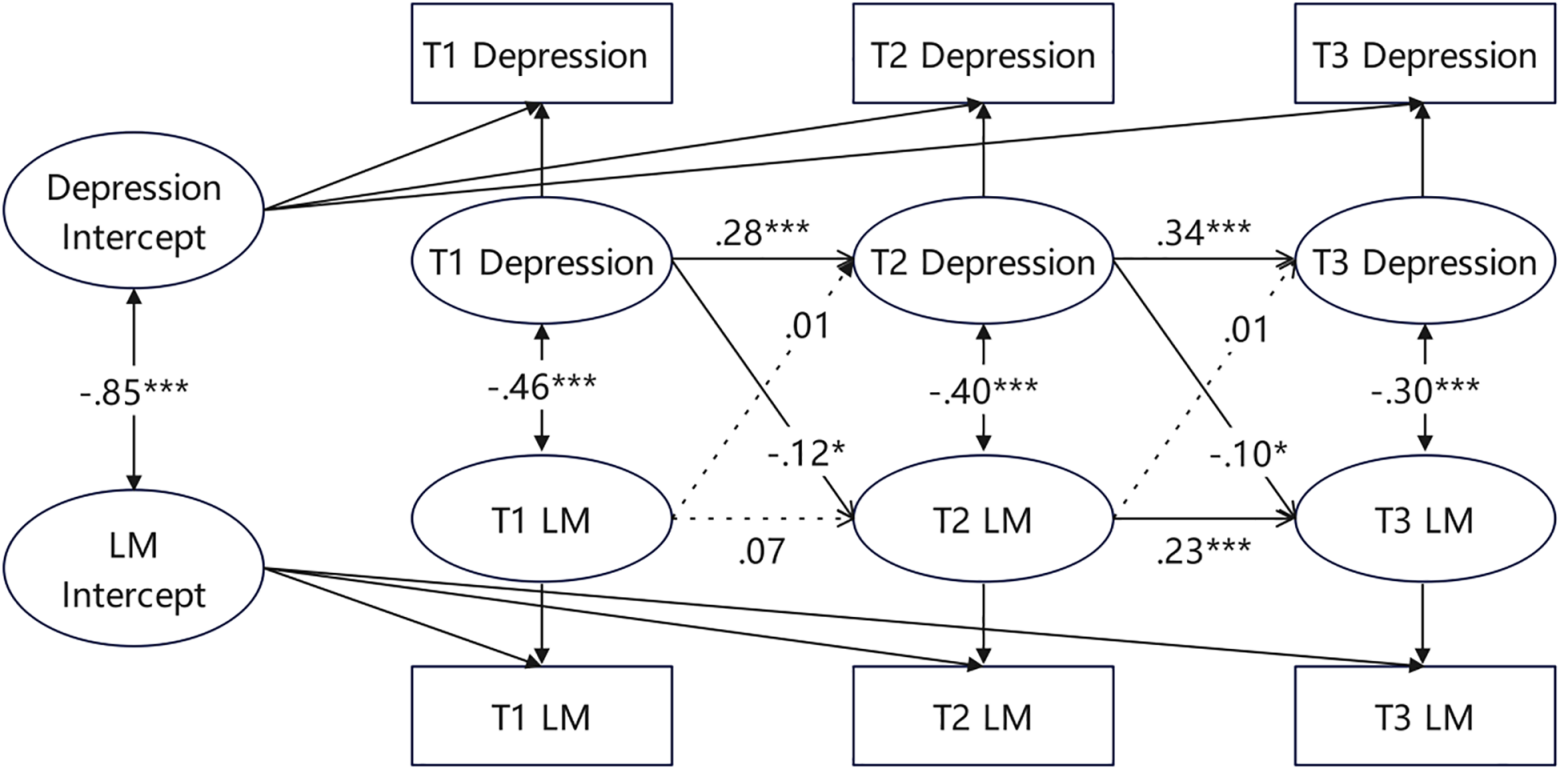
The RI-CLPM of depression and LM across three measurements in families with high educational attainment. Standardized estimates, significant (solid lines) and not significant (dotted lines) paths are included. For the sake of clarity, the effects of the covariates (gender and family economic status) are not shown. *p <.05; ***p <.001.

**Figure 5** shows the RI-CLPM between depressive symptoms and LM in participants from high-educational background families. At the interindividual level, depressive symptoms were negatively correlated with LM (r = -0.85, p < 0.001). At the intraindividual level, depressive symptoms were significantly negatively correlated with LM at all three time points (r = -0.30~-0.46, p < 0.001). Meanwhile, except for the autoregressive effect of LM from T1 to T2, the autoregressive effects of depressive symptoms and LM at all other time points were significant (p < 0.001). In addition, the intraindividual cross-lagged paths from depressive symptoms to LM were significant at T1-T2 (β = -0.12, p < 0.05) and T2-T3 (β = -0.10, p < 0.05). Conversely, no significant cross-lagged effects were observed for the path from LM to depressive symptoms at either T1-T2 or T2-T3. These results suggest that at the intraindividual level, depressive symptoms in high-educational background families still negatively predicted LM across all three time points, whereas LM did not predict depressive symptoms at any time point.

## Discussion

Using three-wave follow-up data from 4,669 college freshmen, this study systematically explored the association pattern between depressive symptoms and LM, and analyzed the potential role of parental educational background in this relationship. The results not only revealed a stable negative correlation between depressive symptoms and LM (LM) at both the interindividual and intraindividual levels but also clarified the dynamic interaction between the two over time through the RI-CLPM model, providing important insights for understanding the mental health and academic adaptation of college freshmen.

### Long-term characteristics at the interindividual level: stable negative correlation

Our results are consistent with previous studies on the relationship between LM and depressive symptoms at the interindividual level (37). RI-CLPM results showed a strong negative correlation between LM and depressive symptoms at the interindividual level (r = -0.70), indicating that individuals with consistently low LM exhibited higher levels of depressive symptoms across the three time points, and vice versa. The strength of this effect was higher than that of concurrent correlations (-0.530~-0.556), suggesting that the interindividual association becomes more prominent after aggregating data from multiple time points. Meanwhile, these interindividual findings highlight the need for targeted interventions, emphasizing that college freshmen with low LM may have higher depressive symptoms, and those with high depressive symptoms may also experience decreased LM.

### Dynamic interaction and autoregressive effects: immediate negative feedback and prediction of sustained deviation

Intraindividual analysis using RI-CLPM further revealed the dynamic interaction between LM and depressive symptoms within individuals. LM and depressive symptoms were significantly negatively correlated at all three time points (r = -0.36~-0.45), indicating that when an individual’s LM deviates from their personal norm at a certain time point, their depressive symptoms deviate in the opposite direction. This result reflects the immediate interaction between LM and depressive symptoms. For example, sudden depressive mood may quickly reduce learning concentration, leading to a temporary decrease in motivation; conversely, a short-term decline in LM may trigger academic frustration, which in turn exacerbates depressive mood, forming a negative feedback loop (8). This immediate association suggests that short-term interventions for college students should address the synchronous fluctuations of both factors, requiring simultaneous provision of academic support and emotional counseling.

The autoregressive effects of both depressive symptoms and LM were significant, indicating that deviations in an individual’s state at a certain time point can predict sustained deviations at subsequent time points. Consistent with our findings, previous studies have shown that past LM exerts a positive impact on future LM. Participants often make emotional predictions about future events; anticipating the feelings after receiving positive feedback can increase their motivation scores in subsequent evaluations (38). In addition, studies have found that a history of depression can significantly predict the occurrence of future depression: depression leads to more negative evaluations of future expectations and increased stress, and this negative thinking in turn predicts subsequent depression (39–41). This finding underscores the importance of early intervention: timely intervention when abnormal fluctuations in LM or depressive symptoms are first observed may prevent the continuation of negative states into the long term.

### Differences in cross-lagged effects: stable prediction of LM by depressive symptoms and stage-specific protective role of LM

At the intraindividual cross-lagged paths, we found that the negative predictive effect of depressive symptoms on subsequent LM was stronger and more stable, while the predictive effect of LM on depressive symptoms varied across families with different educational backgrounds.

Consistent with previous studies, the full-sample analysis showed that depressive symptoms significantly predicted subsequent LM in individuals. This means that when an individual’s depressive symptoms exceed their personal baseline level at a certain time point, their LM at the next time point is more likely to be lower than their personal expectation (42). Existing studies have confirmed that depressive symptoms significantly impair the cognitive abilities of college students, and reducing depressive symptoms can enhance LM by freeing up cognitive resources (43). Specifically, college students with depressive symptoms exhibit impaired semantic inhibition and increased pupillary reactivity during task performance—a phenomenon indicating increased cognitive workload, further confirming the existence of cognitive impairment (44). Such cognitive impairment often makes it difficult for students to concentrate on learning. In addition, low mood and lack of energy reduce their enthusiasm for academic activities, leading to decreased participation and subsequent academic performance decline; this, in turn, exacerbates low self-confidence in a vicious cycle, ultimately negatively affecting LM (8). Other studies have found that individuals with depressive symptoms have increased perceived uncertainty about social outcomes in learning tasks and reduced motivation to participate in real social activities. This state may prompt them to avoid the classroom and campus environment, further weakening LM (45). In summary, depressive symptoms inhibit college students’ LM through multiple pathways; alleviating depressive symptoms is of great significance for improving LM and academic development.

In contrast, regarding the prediction of depressive symptoms by LM, significance was only observed in the T1→T2 stage in the full sample (β = -0.11, p < 0.001), with no significant association observed in the T2→T3 stage. This stage-specific difference requires in-depth analysis in the context of college freshmen’s developmental stages.

The T1 stage coincides with the initial adaptation period after college enrollment—a critical phase during which freshmen need to adapt to new academic models, interpersonal relationships, and self-management. LM is a key psychological resource influencing this adaptation process (46). Studies have shown that LM is directly associated with factors such as self-efficacy, teacher-student relationships, and peer relationships; it not only enhances emotional protection and emotional resilience, supports effective self-management, but also directly affects academic achievement and the ability to cope with the challenges of higher education (47). Self-Determination Theory posits that when individuals feel their needs for autonomy, competence, and relatedness are satisfied in a new environment, intrinsic motivation strengthens psychological resilience and reduces the occurrence of negative emotions (48). Previous studies have also shown that clear directions and challenging goals can predict a reduction in depression (49). Meanwhile, basic learning processes remain effective in depression; motivation deficits may stem from more complex origins rather than impairments in basic learning mechanisms, suggesting that improving LM at the source can reduce depressive levels (50). Therefore, among college freshmen, LM at the T1 stage can effectively predict depressive symptoms at the T2 stage.

After entering the T2→T3 stage, adaptation gradually stabilizes, and the complexity of academic tasks increases; at this point, the predictive effect of LM on depressive symptoms weakens or even disappears. The effect of LM in the T1→T2 stage may reflect the role of “from scratch” basic motivation (e.g., “whether one is willing to engage in learning”), whereas academic demands in the T2→T3 stage rely more on in-depth abilities such as learning strategies, time management, or external support (51). At this stage, simple motivation levels may be insufficient to cope with higher-order academic stress, and their buffering effect on depression is diluted by other more critical adaptive resources.

### Differences in family educational background: initial motivation dependence in low-education families versus multiple supports in high-education families

The subgroup differences in family educational background constitute an important finding of this study. Studies have shown that parental involvement in their children’s education is generally more common among highly educated individuals. Such involvement provides emotional support and resources, which may act as protective factors against depressive emotions (52). In addition, parental motivational characteristics (e.g., courage and growth mindset) can also alleviate depressive symptoms in adolescents (53). A study involving 1,844 students noted that parental and child educational expectations influence depressive symptoms in Chinese adolescents: students with mismatched educational expectations and highly educated parents had significantly higher depressive levels (54). This further suggests that the predictive effect of LM on depressive symptoms is influenced by factors such as parental educational background.

In this study, at the interindividual level, the negative correlation coefficient between depressive symptoms and LM in the intermediate-education family group (r = -0.72) fell between that of the low-education family group (r = -0.66) and the high-education family group (r = -0.85), with all reaching a high effect size. This reflects that the gradient difference in parental educational background may correspond to a gradient in the strength of the association between depressive symptoms and LM.

In terms of intraindividual dynamic predictive paths, among students from low-education families, the predictive effect of LM on depression was only retained in the T1→T2 stage (β = -0.12, p < 0.01). For the intermediate-education family group, LM had a significant predictive effect on depressive symptoms only in the T2→T3 stage (β = 0.08, p < 0.05), with no significant effect observed in the T1→T2 stage. Meanwhile, among students from high-education families, there was no significant effect in all stages. Students from low-educational background families may have limited environmental support or academic guidance provided by their families; thus, they rely more on their own LM as an adaptive resource during the initial enrollment period. Therefore, the buffering effect of T1 LM on T2 depressive symptoms is relatively prominent. However, as time progresses, when academic challenges exceed the coping capacity of their own motivation and families cannot provide additional external support, the protective role of LM becomes difficult to maintain. In contrast, parents from high-educational background families are more likely to be involved in education, providing emotional support, academic guidance, and positive parenting practices that enhance their children’s emotional resilience—making it difficult for fluctuations in LM to translate into depression risk. Notably, parents from high-educational background families may also impose higher educational expectations on their children; this pressure may also weaken the protective role of LM, resulting in no significant predictive effect of LM on depressive symptoms at any stage. However, students from families with intermediate educational backgrounds may rely on both family support and campus adaptation resources. In the T1→T2 stage, fluctuations in LM had not yet become a core factor affecting depressive symptoms. After entering the T2→T3 stage, the support provided by families gradually became insufficient to cope with complex academic challenges. At this point, the strength of LM began to directly affect students’ emotional states and might even intensify academic frustration, thereby contributing to depressive emotions.

The effect sizes of the β values (-0.10 to -0.23) for the cross-lagged paths in this study are small, which may be influenced by the large sample size. Despite statistical significance, the association strength is weak. However, for early interventions at the population level, even small effects may accumulate to significant public health value. Our findings not only provide longitudinal evidence for understanding the interaction mechanism between college students’ mental health and academic issues but also lay an empirical foundation for the development of stratified intervention strategies in colleges and universities. Given the cross-temporal predictive effect of depressive symptoms on LM, colleges and universities should prioritize depression screening and early intervention to prevent the continuous erosion of LM. Interventions targeting LM should consider family background: for students from low-educational background families, simultaneous attention should be paid to improving LM and providing emotional support; external support (e.g., teacher or peer assistance) can be used to supplement insufficient family support, preventing a decline in motivation from triggering depressive symptoms. For students from high-educational background families, focusing on alleviating depressive symptoms may be more effective, as their depressive symptoms are less likely to be influenced by LM. In addition, colleges and universities should establish a mechanism for early warning and rapid intervention. They should identify students with abnormal LM or depressive symptoms in the early stage of enrollment, implement peer mentoring programs, and block the continuation of negative states through short-term counseling. During the entire college period, they should increase training in learning strategies and time management, and provide targeted academic or psychological support channels, especially for students from families with low or intermediate educational attainment.

## Limitations

This study is innovative in that it used three-wave longitudinal data to investigate the relationship between depressive symptoms and LM among college freshmen and discussed the subgroup analysis of family educational background. Meanwhile, interindividual and intraindividual effects were analyzed separately during the analysis process. However, the study has several limitations. First, the sample only included freshmen from a single university; thus, the results may not be generalizable to students of other grades, from other regions, or to non-college student populations. Second, variable measurement relied on self-reports, which may be subject to subjective bias; future studies could combine teacher evaluations and physiological indicators to improve data robustness. Third, the time span of the three-wave data is limited, and long-term dynamic associations still require verification through longer follow-up periods. Future studies could further expand sample diversity and introduce experimental intervention designs to directly test “whether alleviating depression can improve LM” and “whether improving LM has equivalent effects on reducing depression in students from different family backgrounds,” providing more robust evidence for causal inference. Fourth, confounding factors such as academic major, academic performance, personality traits, perceived stress levels, lifestyle factors unrelated to learning, medical or psychiatric history, and family structure were not adjusted for.

## Conclusion

Using three-wave longitudinal data and the RI-CLPM model, this study systematically revealed the longitudinal association between depressive symptoms and LM among college freshmen, as well as subgroup differences related to family educational background. The main conclusions are as follows:

Depressive symptoms and LM showed a stable negative correlation both at the interindividual and intraindividual levels, forming a stable interindividual difference and an immediate intraindividual vicious cycle of “lower motivation leads to more severe depression, and more severe depression leads to lower motivation.”

Abnormal states of depression or motivation persist over time; early intervention can block the negative cycle.

Cross-lagged effects showed asymmetry: the negative predictive effect of depressive symptoms on subsequent LM was stronger and stable across stages, whereas the predictive effect of LM on depressive symptoms was stage-specific.

There are subgroup differences in the association between depressive symptoms and LM across family educational backgrounds. Students from low-education families initially rely on LM to buffer depressive symptoms, but this effect is difficult to maintain in the later stage due to insufficient support. For students from high-education families, the predictive effect of LM on depressive symptoms is not significant, which may be attributed to more adequate parental support or the pressure from parents’ educational expectations. As for students from families with intermediate educational backgrounds, they may rely on both family support and campus adaptation resources simultaneously.

## Data Availability

The raw data supporting the conclusions of this article will be made available by the authors, without undue reservation.
